# miR-199b-3p contributes to acquired resistance to cetuximab in colorectal cancer by targeting CRIM1 via Wnt/β-catenin signaling

**DOI:** 10.1186/s12935-022-02460-x

**Published:** 2022-01-28

**Authors:** Hu Han, Yan Li, Wan Qin, Lu Wang, Han Yin, Beibei Su, Xianglin Yuan

**Affiliations:** 1 Department of Oncology, Tongji Hospital, Huazhong University of Science and Technology, No. 1095 Jiefang Avenue, Wuhan, 430030 Hubei China; 2 Department of Oncology, First Affiliated Hospital, School of Medicine, Shihezi University, Shihezi, Xinjiang China; 3 Medical Department, First Affiliated Hospital, School of Medicine, Shihezi University, Shihezi, Xinjiang China

**Keywords:** Colorectal cancer, Cetuximab, microRNA, Chemoresistance, Wnt/β-catenin signaling, Therapeutic target

## Abstract

**Background:**

Despite advances in the development of efficient chemotherapy, the treatment of colorectal cancer (CRC) remains a challenge due to acquired chemoresistance. It has been reported that microRNAs (miRNAs) dysregulation is associated with the development of chemoresistance. Recently, the expression of miR-199b-3p has been found to be significantly different between cetuximab (CTx)-resistant and -sensitive CRC cells. However, its role and the underlying mechanisms in acquired chemoresistance to CTx in CRC are still obscure.

**Methods:**

Here we report that miR-199b-3p is significantly up-regulated in both CTx-resistant (CTxR) CRC tissues and cell lines.

**Results:**

Functional assays showed that suppressing miR-199b-3p could improve the sensitivity of CRC-CTxR cells to CTx, thereby reducing cell proliferation, migration and invasion, and enhancing cell apoptosis. Mechanistic studies revealed that CRIM1 is a direct target of miR-199b-3p in CRC-CTxR cells; and the effect of miR-199b-3p on CTx-resistance was exerted by regulating the Wnt/β-catenin signaling pathway via CRIM1. Furthermore, mice xenograft models were established and confirmed that down-regulating miR-199b-3p restores the inhibition effect of CTx on tumor growth in CRC-CTxR. Collectively, our data suggest that silencing miR-199b-3p could enhance the anti-tumor effects of CTx on CTx-resistant CRC in vitro and in vivo by activating Wnt/β-catenin signaling via the down-regulation of CRIM1.

**Conclusions:**

Our findings suggest miR-199b-3p might serve as a promising therapeutic target against CTx resistant CRC, and provide scientific information for exploring novel strategies of improving the efficacy of CTx for CRC patients.

**Supplementary Information:**

The online version contains supplementary material available at 10.1186/s12935-022-02460-x.

## Background

Colorectal cancer (CRC) ranks as the second leading cause of death from common malignancies, second only to lung cancer [1]. With the development of modern medical technology, screening and early diagnosis has greatly reduced the mortality of CRC in the last few decades [2]. Meanwhile, adjuvant chemotherapy has also achieved significant effects in CRC patients with lymph node metastasis [3]. Cetuximab (CTx), an epidermal growth factor receptor (EGFR) inhibitor, is approved by FDA for the first-line therapy for EGFR-expressing metastatic CRC with wild-type RAS, which competitively blocks the EGFR extracellular domain to inhibit the phosphorylation of EGFR thus suppressing the proliferation and metastasis of CRC cells [4, 5]. However, acquired resistance to CTx occurs in the majority of CRC patients after a period of treatment and eventually results in CRC recurrence [6–8]. Thus, it’s urgent to develop novel therapeutics for overcoming chemoresistance and improving the efficacy of chemotherapy in patients with CRC.

Over the past decades, microRNAs (miRNAs) have been gaining prominence in various disease fields due to their ability to modulate gene expression at the post-translation level [9]. Increasing studies documented that miRNAs are involved in regulating chemoresistance in CRC, such as miR-637 [10], miR-302a [11], and miR-196b-5p [12]. miR-302a regulates the expression of both NFIB and CD44 to suppress metastasis and CTx resistance in CRC [11]. Ren et al. [12] demonstrated that down-regulating miR-196b-5p could restore the 5-fluorouracil responsiveness of CRC cells through impairing cancer stemness. miR-199 is one of the most important miRNA families, which has been implicated in various tumors as either promoters or suppressors [13]. There are two members in the miR-199 family, which include miR-199a and miR-199b. Nowadays, researchers mainly focus on the biological function of miR-199a; and the roles of miR-199b are largely unknown. Mussnich et al. [14] identified both miR-199a-5p and miR-199b-3p as significant differentially expressed miRNAs between CTx-sensitive and CTx-resistant CRC cells. They also further demonstrated that miR-199a-5p contributed to acquired resistance to CTx in CRC cells by conducting functional analyses. While the expression of miR-199b-3p is functionally involved in acquired resistance to CTx in CRC has not been reported yet.

As reported, the molecular mechanisms of acquired resistance to CTx in CRC involve two main signaling pathways: EGFR signaling and Wnt signaling. EGFR mainly transmits signals from the cytoplasm to the nucleus through two pathways, namely Ras/Raf/MEK/ERK/MAPK and PI3K/AKT/mTOR. Abnormalities in any relevant molecules of these pathways may lead to the development of CTx resistance [15, 16]. As an important pathway for maintaining cell homeostasis and embryonic development, the Wnt signaling is evolutionarily conserved, which has been demonstrated to be associated with cancer apoptosis, epithelial-mesenchymal transition (EMT), stemness, and tumor microenvironment [17]. The most well-studied Wnt pathway is the Wnt/β-catenin signal transduction, which also refers to the canonical Wnt pathway. In the absence of Wnt signaling, β-catenin is degraded by a protein complex of Axin, adenomatous polyposis coli (APC), and glycogen synthase kinase 3β (GSK3β). The activation of Wnt signaling protects β-catenin from phosphorylation by the protein complex. Then, non-phosphorylated β-catenin accumulates in the cytoplasm and transfers to the nucleus, followed by binding to T-cell factor/lymphoid enhancer binding factors (TCF/LEF) to regulate several oncogenes, thereby promoting tumor cell proliferation and reducing apoptosis [18, 19]. Mounting evidence signposted that the Wnt/β-catenin activity level positively correlates with the chemoresistance to several drugs in CRC [20, 21], including CTx [22, 23]. A previous study demonstrated that miR-199b is involved in the regulation of Wnt/β-catenin signaling of cisplatin resistance in CRC stem cells [24]. Therefore, our study speculated that miR-199b-3p contributes to CTx-resistance in CRC via regulating Wnt/β-catenin signaling.

The present study found that miR-199b-3p is significantly up-regulated in CRC tissues and cells with CTx-resistance and down-regulating miR-199b-3p could re-sensitize chemoresistant CRC cells to CTx in vitro and in vivo. Combing bioinformatics analysis and in vitro experiments, CRIM1 was identified as a direct target of miR-199b-3p. In addition, the biological role and mechanism of miR-199b-3p/CRIM1 in CRC chemoresistance was further explored. Collectively, our study provided evidence showing that the regulatory role of miR-199b-3p in acquired resistance to CTx in CRC was mediated by CRIM1 via the Wnt/β-catenin signaling pathway.

## Methods

### Clinical samples collection

Thirty primary tumor tissue samples were collected from 30 CRC patients who received CTx chemotherapy in Tongji Hospital, Huazhong University of Science and Technology. The included patients had undergone at least two cycles CTx therapy (CTx 500 mg/m^2^ i.v over 2 h first infusion, then 250 mg/m^2^ i.v over 60 min every 2 weeks), and were evaluated for a tumor response at a minimum of every 4 weeks while on CTx therapy. Patients who experience unacceptable toxicity or who have progressive disease will not receive further CTx therapy. According to Response Evaluation Criteria in Solid Tumors (RECIST) criteria [25], these patients were classified into responders (n = 15; patients with CR (complete response) or PR (partial response)) and non-responders (n = 15; patients with SD (stable disease) and PD (progressive disease)). The clinicopathological characteristics of the patients are summarized in Table 1. Written consent was obtained from all participants (or their guardians), and the procedure of this study was carried out following the Chinese Ethical Regulations and Guidelines approved by the Research Ethics Committee of Tongji Hospital, Huazhong University of Science and Technology.

**Table 1:** Characteristics of colorectal cancer patients with CTx therapy

	All patients n (%)	Responders n (%)	Non-responders n (%)	*p*-value*
Total number	30	15	15	-
Gender				0.7150
Male	15	7	8	
Female	15	8	7	
Age				0.1953
< 60	7	2	5	
≥ 60	23	13	10	
Median (range)	67 (44–83)			
Tumor grade				0.3091
I–II	29	14	15	
III-IV	1	1	0	
Primary tumor site				0.6242
Colon	6	3	2	
Rectum	24	12	13	

### Immunohistochemistry (IHC) analysis

In brief, paraffin-embedded tissue sections were dewaxed, and rehydrated, followed by incubation with primary antibodies against different proteins, including CRIM1 (ab272542; Abcam, MA, USA), Ki-67 (ab15580; Abcam, MA, USA), and MACC1 (PA5-115530; Thermo Scientific, MA, USA). After that, the sections were incubated with Goat Anti-Human IgG H&L (HRP) preadsorbed (ab7153) or Goat Anti-Mouse IgG + IgM H&L (HRP) preadsorbed (ab47827) secondary antibodies (Abcam, MA, USA) to visualize primary antibody-antigen complexes. Finally, the sections were stained with diaminobenzidine and hematoxylin, and subsequently visualized under the BX-51 microscope. The detailed information of antibodies used in this study was listed in Additional file 5: Table S3.

### Quantitative real-time PCR (qRT-PCR)

Total RNA was extracted from tissue samples and cultured cells using TRIzol reagent (Invitrogen, CA, USA). The cDNA of CRIM1 was synthesized by using a High Capacity cDNA Reverse Transcription Kit (Applied Biosystems, CA, USA). For quantification of miR-199b-3p and CRIM1 based on quantitative realtime PCR was performed using TaqMan miRNA assay probes (Applied Biosystems, CA, USA) and SYBR Premix Ex Taq™ (Takara, Japan). All reactions were run three times. The expression level of miR-199b-3p was normalized to U6, while that of CRIM1 was normalized to β-actin using the 2^–ΔΔCt^ approach [26]. The responder group was used as the reference group. The primers and the corresponding concentration used in this study were as follows: miR-199b-3p (forward (F): 5′-CCAGAGGACACCTCCACTCC-3′, reverse (R): 5′-GGGCTGGGTTAGACCCTCGG-3′), 200 nM; CRIM1 (F: 5′-TTCGGGATTTACGGAACCTGC-3′, R: 5′-GGTGTTACATTCACATTTCCCA-3′), 200 nM; U6 (F: 5′-GCTTCGGCAGCACATATACTAAAAT-3′, R: 5′-CGCTTCACGAATTTGCGTGTCAT-3′), 400 nM; β-actin (F: 5′-AAGGTGACAGCAGTCGGTT-3′, R: 5′-TGTGTGGACTTGGGAGAGG-3′), 150 nM.

### Cell culture and establishment of CTx-resistant SW480/HCT116 cell lines (SW480-CTxR/HCT116-CTxR)

Four human CRC cell lines (SW480, HCT116, HT29, and CaCO_2_) were purchased from ATCC (VA, USA). All these four cell lines were derived from primary tumors of CRC, SW480, HCT116, and HT29 cell lines show high expression of EGFR while CaCO_2_ cell line shows moderate expression. All cells were maintained in RPMI 1640 medium (Gibco, NY, USA) supplemented with 10% FBS (Gibco, NY, USA) and 1% penicillin–streptomycin (Gibco, NY, USA) in a 5% CO_2_ incubator at 37℃.

CRC-CTxR cell lines including SW480-CTxR and HCT116-CTxR were established as Troiani et al. [27] previously reported. Briefly, parental CTx sensitivity CRC cell lines (SW480 and HCT116) were exposed to the intermittently and gradually increasing concentration (1, 5, 10, and 20 μM) of CTx for the duration of more than 6 months. Initially, exposed parental CRC cells to 1 μM CTx, and the surviving population of cells was grown and passaged for 6 weeks. The cells that survived in CTx treatment were then exposed to a higher concentration of CTx for another 6 weeks. In order to keep the CTx resistance of the CRC-CTxR cell lines, a low dose of CTx (2 μM) was added to the culture medium until 1 month prior to the beginning of the study.

### Cell viability assay

CCK-8 kit (Dojindo, Japan) was exploited to detect cell viability. Briefly, cells were seeded in 96-well plates and then treated with different concentrations of CTx in accordance with our study design. Upon the end of treatment (24 h treatment with CTx), CCK-8 reagent was added into each well at a 1:10 ratio of the culture medium and incubated for 1.5 h at 37 °C. After incubation, optical density (OD) was measured at 450 nm under a microplate reader (Thermo Scientific, MA, USA). The IC50 was determined by fitting the concentration vs. inhibition curve based on the function of GraphPad Prism (USA).

### Cell transfection

The miR-199b-3p mimic and miR-199b-3p inhibitor and their corresponding negative control (NC mimic and NC inhibitor) were obtained from GenePharma (Shanghai, China). CRIM1-overexpressing vector (CRIM1 OE), and negative control pcDNA3.1 (Vector) were all supplied by RiboBio (Guangzhou, China). A total of 5 × 10^4^ cells were seeded in 12-well plates and incubated overnight at 37 °C, followed by transfected with the above-indicated plasmids using Lipofectamine® 2000 reagent (Invitrogen, OR, USA) for 48 h. The final concentrations of miR-199b-3p mimic/NC mimic and miR-199b-3p inhibitor/NC inhibitor were 50 and 100 nM, respectively. Cells were transfected with CRIM1-overexpressing vector at a concentration of 1.6 µg/well in a 12-well plate. Thereafter, cells were harvested for further analysis, whose transfection efficiency was confirmed by qRT-PCR.

### Cell apoptosis assay

Terminal deoxynucleotidyl transferase (TdT) dUTP Nick-End Labeling (TUNEL) assay was performed to detect apoptotic DNA fragmentation, thereby identifying apoptotic cells. In brief, the treated cells were fixed in 4% paraformaldehyde for 20 min and permeabilized with Triton X-100 (0.25% in PBS) for another 20 min, followed by washed twice and incubated with TdT reaction buffer for 10 min. Then, the cells were stained using an In Situ Apoptosis Detection Kit (Takara, Japan) and incubated in a humidified chamber at 37 °C. After being washed thrice with 3% BSA in PBS, the cell nuclei were counter-stained with DAPI for 15 min. Finally, TUNEL-positive cells were counted in six random fields for each well under a fluorescence microscope. The percentage of apoptosis was expressed as the percentage of TUNEL-positive cells out of the total number of cells (DAPI-staining cells).

### Western blot

The collected tissues and treated cells were lysed in RIPA buffer (Abcam, MA, USA) on ice for 40 min to extract total protein. After being quantified with BCA kit (ab102536; Abcam, MA, USA), 50 μg of total protein was separated by 12% SDS-PAGE gels and electrophoretically transferred onto PVDF membranes, which were subsequently blocked in skimmed milk (8%) for 2 h. The membranes were incubated overnight at 4 ℃ with primary antibodies against Bax (ab32503; Abcam, MA, USA), Bcl-2 (ab32124; Abcam, MA, USA), CRIM1 (PA5-34,410; Thermo Scientific, MA, USA), ERK1/2 (ab17942; Abcam, MA, USA), T202/Y204 phosphorylated-ERK1/2 (p-ERK1/2) (ab214362; Abcam, MA, USA), Axin2 (ab109307; Abcam, MA, USA), β-catenin (ab32572; Abcam, MA, USA), and β-actin (ab8226; Abcam, MA, USA), followed by rinsed thrice with NaCl/Tris (TBS) supplemented with 0.1% Tween 20 (TBS-T) before the 2 h incubation of secondary antibody of Goat Anti-Rabbit IgG H&L (HRP) (ab6721; Abcam, MA, USA). Finally, bands were visualized by ECL Substrate Kit (Abcam, MA, USA), and quantified using Image J software (USA).

### Cell migration and invasion assay

To explore the migration and invasive potential of CRC cells, the migration and invasion of cells were detected following treatments. The cell migration was assessed by wound healing assay. Briefly, cells were seeded into six-well plates and cultured until a monolayer of cells had formed. The 200-μL pipette tip was used to generate the similar size of scratches in the cell layer for each group. Next, scratched cells were removed by gently rinsing PBS thrice; the remaining cells were continually cultured in serum-free media for 24 h. After scratching for 0 and 24 h, the wound of each well was photographed with the BX51 microscope; and the migrated area was calculated.

The invasion of cells was investigated by Transwell chambers with Matrigel-coated membrane (Corning, NY, USA). In brief, 700 μL DMEM with serum was added to the lower chamber. In the meantime, transfected cells were plated on the upper chambers containing 200 μL DMEM for 24 h incubation. 24 h later, cells still in the upper chamber were wiped out, while cells traversing the membranes to the lower chamber were fixed in 4% paraformaldehyde prior to the staining of 0.1% crystal violet. Non-invaded cells were wiped with a cotton swab, and five randomly selected fields were imaged and counted per chamber under a BX51 microscope (Magnification, × 100).

### Bioinformatics analysis

GSE140973 (https://www.ncbi.nlm.nih.gov/geo/query/acc.cgi?acc=GSE140973), a dataset containing the mRNA expression profiling of colon cancer xenograft model exhibiting response and secondary resistance to CTx treatment (non-CTx resistance and CTx resistance groups), was downloaded by the GEO database. The differentially expressed genes (DEGs) between non-CTx resistance and CTx resistance groups were identified using the limma R package [28] with a threshold of P < 0.05 and |log2 Fold change (FC)|≥ 1.

The potential target genes of miR-199b-3p and the corresponding binding sites were predicted by Targetscan (http://www.targetscan.org/vert_72/) [29]. The genes obtained by intersecting identified down-regulated genes and predicted target genes of miR-199b-3p were selected for further analysis.

### Dual-luciferase assay

The CRIM1 fragments containing the binding site for miR-199b-3p were cloned into the luciferase reporter vector (Promega, WI, USA) (CRIM1 WT). Mutation (MUT) sites were also designed (CRIM1 MUT). Cells were transferred into 24-well plates at 3 × 10^4^ cells per well. After 24 h, the cells were transiently co-transfected with 0.1 μg/well of the luciferase reporter vector (CRIM1 WT or CRIM1 MUT) and 50 nM miR-199b-3p mimic/NC mimic into SW480-CTxR/HCT116-CTxR cells using Lipofectamine® 2000 reagent for 48 h, the cells were collected to test the activities of firefly and Renilla luciferase by the Dual-Luciferase Assay System (Promega, WI, USA). Relative luciferase activity was expressed as the ratio of Renilla/firefly.

### Fluorescence in situ hybridization (FISH)

The subcellular localization of miR-199b-3p was identified with FISH. Briefly, cells were seeded on glass slides and fixed with 4% paraformaldehyde. After permeabilization with 0.25% Triton X-100 and washing with sodium citrate buffer, the cells were stained using FITC-labelled probes specific for miR-199b-3p (Ribo, Guangzhou, China) in a hybridization buffer at 37 °C overnight. Next, DAPI was used for cell nucleus counterstain. Finally, the cells were rinsed three times, followed by observation under a fluorescence microscope (BX-51; Olympus, Japan).

### RNA immunoprecipitation (RIP) assay

The RIP assay was conducted by using a Magna RIP Kit (Millipore, MA, USA) according to the guidelines provided by manufacturers. For anti-Ago2 RIP, cell extract was immunoprecipitated with magnetic beads conjugated with antibodies against Ago2 (ab32381; Abcam, MA, USA). Next, miR-199b-3p and CRIM1 that immunoprecipitated by Ago2 were analyzed by qRT-PCR. IgG was served as a negative control.

### Animal experiments

Twenty BALB/c nude mice (4-5 weeks, male) were purchased from Guangdong Medical Laboratory Center (Guangzhou, China). Mice were randomized into four groups (n = 5), and subcutaneously injected with 0.1 mL PBS containing 1.5 × 10^6^ SW480-CTxR cells to establish the xenograft model. On the 8th day post-inoculation, mice in four groups received different treatments twice a week as followed: saline group (mice intraperitoneally injected with 0.1 mL saline), CTx group (mice intraperitoneally injected with 0.1 mL CTx (2 mg/kg) [30]), miR-199b-3p antagomir group (mice intraperitoneally injected with 0.1 mL specific antagomiR directed against miR-199b-3p (150 μg; AcceGen, NJ, USA), CTx + miR-199b-3p antagomir group (mice intraperitoneally injected with the combination of CTx and miR-199b-3p antagomir). After cell inoculation for a week, the growth of established xenografts on each mouse was monitored using a Vernier caliper every five days (days 7, 12, 17, 22, 27, 32, and 37) and calculated based on the formula: length × width^2^/2. On the 37th day post-inoculation, mice were sacrificed by cervical dislocation to collect xenografts for weighing and the IHC analysis. All applicable international, national, and/or institutional guidelines for the care and use of animals were followed. This study was approved by Tongji Hospital, Huazhong University of Science and Technology.

### Statistical analysis

All experiments were performed thrice at least. Data are expressed as mean ± SD. GraphPad Prism was applied to conduct statistical analysis of all data. The comparisons among groups were performed using student’s t-test or one-way ANOVA followed by Bonferroni’s post hoc test. It is considered to be statistically significant when P < 0.05.

## Results

### miR-199b-3p expression is positively associated with acquired resistance to CTx in CRC

**Fig. 1:**
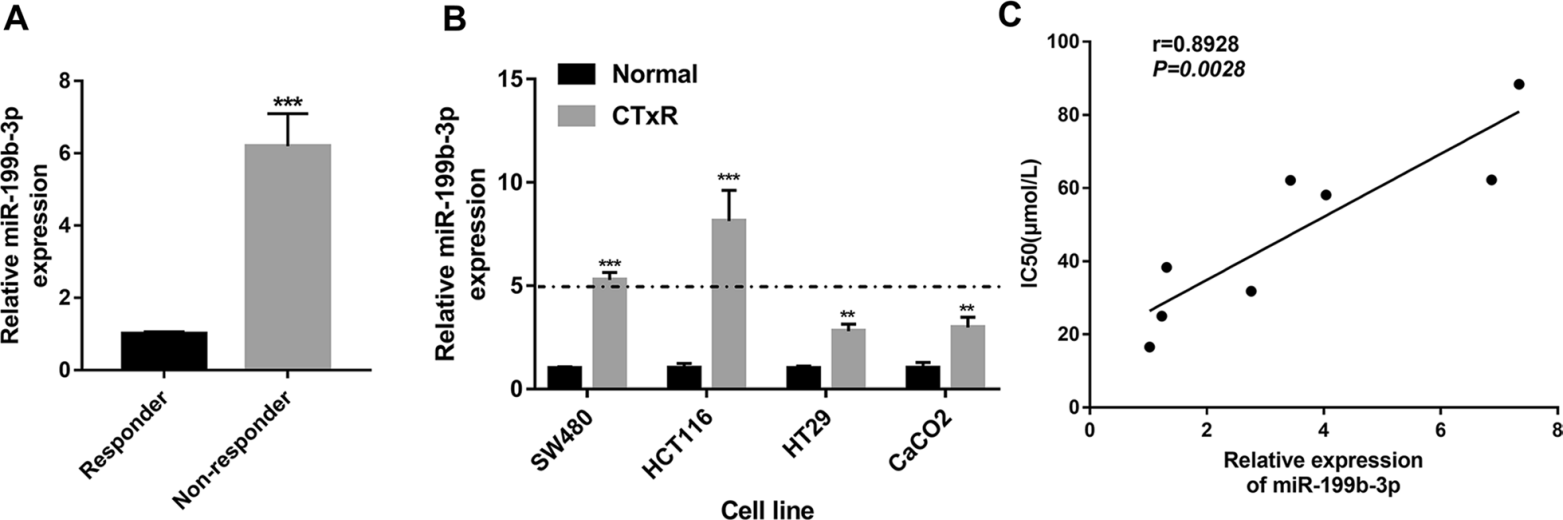
miR-199b-3p is highly expressed in the CTx-resistant CRC tissues and cells. **A** The expression of miR-199b-3p in CRC tissues between responder and non-responder groups (n = 15 per group). **B** The expression of miR-199b-3p between CRC-CTxR cell lines and the parental sensitive ones. **C** The correlation between the expression level of miR-199b-3p and IC50 for CTx in eight CRC cell lines (SW480-CTxR, HCT116-CTxR, HT29-CTxR, CaCO2-CTxR, SW480, HCT116, HT29, and CaCO2). ^**^P < 0.01 and ^***^P < 0.005 compared with responder or normal group

Initially, qRT-PCR detecting the expression levels of miR-199b-3p in the CRC tissues showed that miR-199b-3p is significantly up-regulated in CTx-non-responders compared with CTx-responders (Fig. 1A). Differential expressions of miR-199b-3p, which may be associated with chemoresistance, were detected in CRC-CTxR cell lines compared with parental sensitive ones (Fig. 1B). The data showed that miR-199b-3p was significantly up-regulated more significantly in SW480-CTxR and HCT116-CTxR cells (> fivefold change), compared with HT29-CTxR and CaCO2-CTxR cells (Fig. 1B). Hence, SW480-CTxR and HCT116-CTxR cells were selected for the subsequent functional analysis. Meanwhile, expression profiles of EGFR in CRC-CTxR and the parental sensitive cell lines were also analyzed, which showed the expression level of EGFR in different CRC cell lines was decreased under the condition of chemoresistance (Additional file 1: Fig. S1). Additionally, the Pearson correlation analysis showed a significant positive correlation between the miR-199b-3p level and CTx resistance (half-maximal inhibitory concentration (IC50)) in these eight CRC cell lines (Fig. 1C).

### miR-199b-3p contributes to the CTx-resistance of CRC cells

**Fig. 2:**
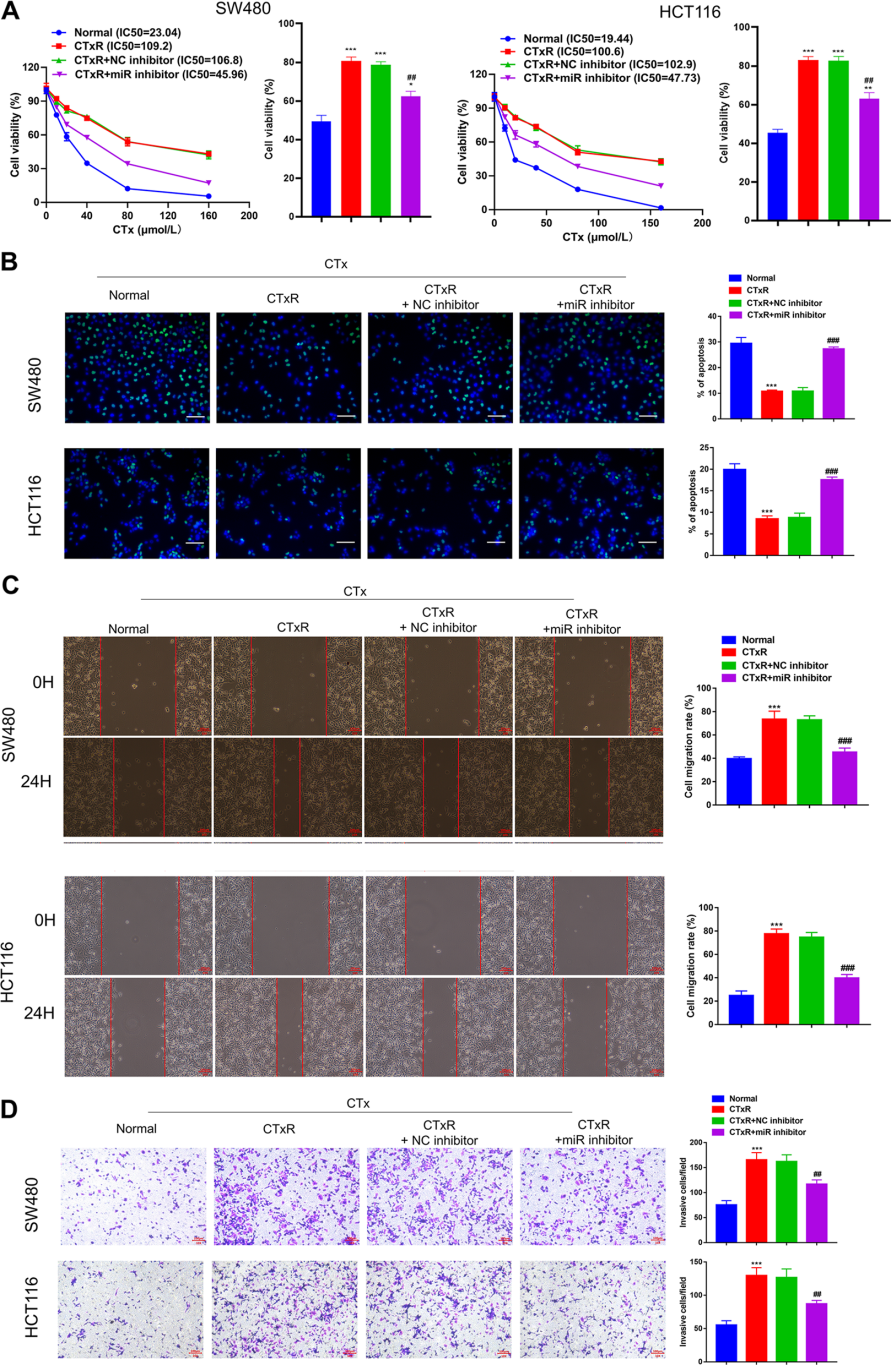
miR-199b-3p contributes to acquired resistance to CTx in CRC cells. CRC-CTxR cells (CTxR) were transfected with miR-199b-3p inhibitor (miR inhibitor) or NC inhibitor; then, CRC-CTxR cells with or without transfection and the parental sensitive ones (normal) were treated with the same concentration of CTx. **A** CCK-8 assay analyzed the IC50 of CTx in CRC cells. **B** TUNEL assay detected the apoptotic cells of different groups. **C** Wound healing assay assessed cell migrative ability. **D** Transwell assay detected the capacity of cell invasion. (Scale bars: 100 μm). *P < 0.05, **P < 0.01, and ***P < 0.005 compared with normal group; ^##^P < 0.01 and ^###^P < 0.005 compared with CTxR group

qRT-PCR analysis exhibited that miR-199b-3p inhibitor successfully down-regulated the expression of miR-199b-3p in both SW480-CTxR and HCT116-CTxR cells (Additional file 1: Fig. S1B). The growth curves showed that the IC50 of CTx in CRC-CTxR cells was dramatically higher than that in parental sensitive ones (Fig. 2A), hinting the successful establishments of SW480-CTxR and HCT116-CTx. In the meantime, silencing miR-199b-3p could rescue the CTx responsiveness of SW480-CTxR and HCT116-CTxR (Fig. 2A). Apoptosis is another important indicator of the antineoplastic effects of CTx. The apoptosis of CRC cells was measured by TUNEL assay and western blot (detecting apoptosis-related proteins) to investigate the role of miR-199b-3p in CTx-induced apoptosis. As expected, under the treatment of CTx, the apoptosis rate of CRC-CTxR cells was significantly lower than that of parental sensitive ones (Fig. 2B). Besides, the results showed that miR-199b-3p inhibitor accelerated the apoptosis of CRC-CTxR cells (Fig. 2B). In addition, western blot showed that CTx increased the Bax/Bcl-2 ratio in CTx sensitive CRC cells, but this effect was impaired in CRC-CTxR cells (Additional file 1: Fig. S1A). After knocking down miR-199b-3p in CRC-CTxR cells, Bax/Bcl-2 levels were significantly increased, which suggested that miR-199b-3p inhibitor could restore the sensitivity of CRC-CTxR cells to CTx (Additional file 1: Fig. S1A). Given that CTx is the first-line drug for metastatic CRC, we also carried out wound healing and transwell invasion assay to investigate the migration and invasion abilities of CRC cells in vitro under different contexts. Under the treatment of CTx, the migration and invasion abilities of CRC-CTxR cells are significantly stronger than those of parental sensitive ones, which could be reduced by miR-199b-3p inhibitor (Fig. 2C, D). Collectively, suppressing miR-199b-3p may enhance the sensitivity of CRC-CTxR cells to CTx, thereby promoting the effect of CTx on proliferation, apoptosis, migration, as well as invasion of CRC-CTxR cells.

### Identification of CRIM1 as a direct target of miR-199b-3p in acquired resistance to CTx in CRC

**Fig. 3:**
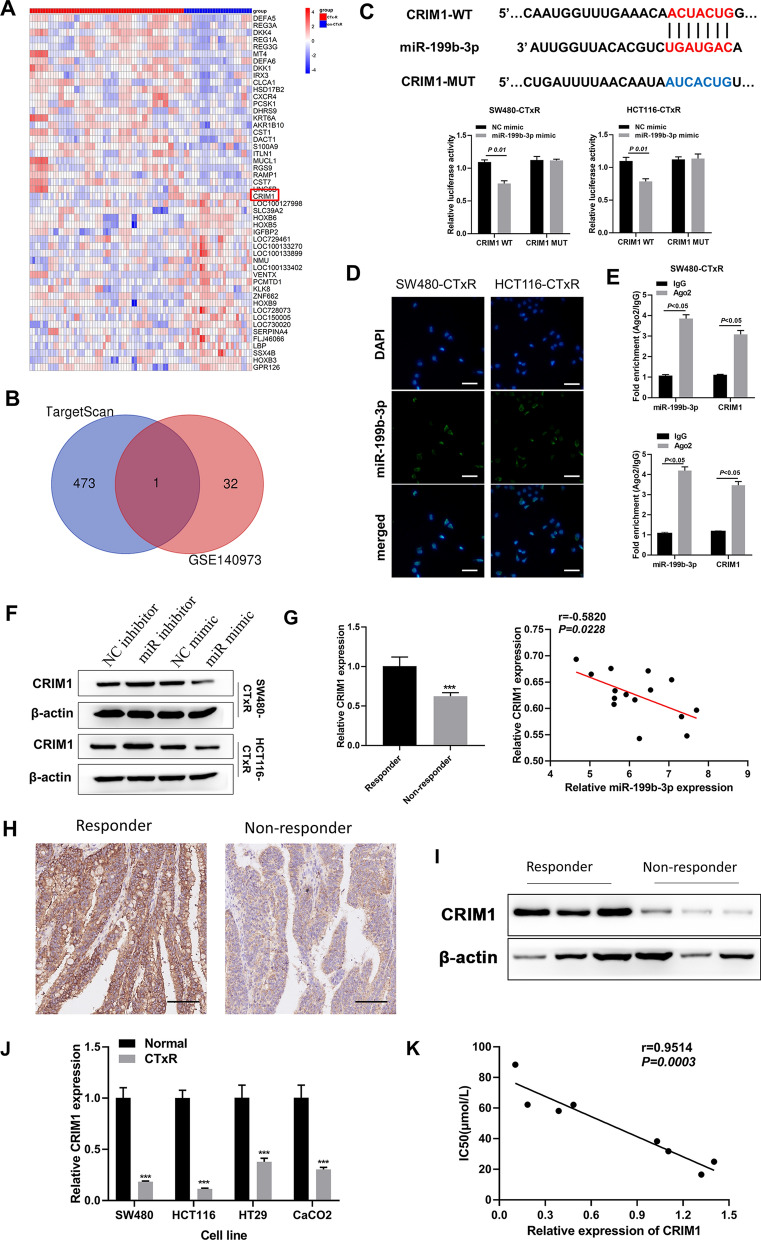
CRIM1 is a direct target of miR-199b-3p in acquired resistance to CTx in CRC. **A** Heatmap of the top 25 up-regulated genes and down-regulated genes between CTx treated tumors and CTx secondary resistant tumors based on GSE140973 dataset. **B** Venn diagram displaying only one down-regulated gene (CRIM1) of CTx secondary resistant tumors in the list of potential targets of miR-199b-3p. **C** Dual-luciferase reporter assay confirmed the interaction between miR-199b-3p and CRIM1 in SW480-CTxR and HCT116-CTxR (The putative binding sites were predicted using targetscan). **D** FISH assay displayed the intracellular distribution of miR-199b-3p in SW480-CTxR and HCT116-CTxR (Scale bars: 50 μm). **E** Anti-Ago2 RIP assay followed by qRT-PCR determined the association between miR-199b-3p and CRIM1 with Ago2 in SW480-CTxR and HCT116-CTxR. **F** Western blot examined the protein expression levels of CRIM1 in SW480-CTxR and HCT116-CTxR after the transfection of miR-199b-3p inhibitor or mimic. **G** The expression of CRIM1 in CRC tissues between responder and non-responder groups (n = 15 per group) using qRT-PCR (left); Pearson correlation analysis for miR-199b-3p and CRIM1 expression in CRC tissues from non-responders (n = 15) (right). **H** Representative images of CRIM1 expression in CRC tissues between responder (high expression) and non-responder (low expression) using IHC (Scale bars: 100 μm). **I** Western blot examined the protein expression levels of CRIM1 in CRC tissues between responder and non-responder groups. **J** The expression of CRIM1 between CRC-CTxR cell lines and the parental sensitive ones. **K** The correlation between the expression level of CRIM1 and IC50 for CTx in eight CRC cell lines (SW480-CTxR, HCT116-CTxR, HT29-CTxR, CaCO2-CTxR, SW480, HCT116, HT29, and CaCO2). ***P < 0.005 compared with responder or normal group

To explore the potential mechanism of miR-199b-3p for acquired resistance to CTx in CRC, we performed a series of bioinformatics analyses to identify the target gene of miR-199b-3p in CTx-resistant CRC. By performing differential expression analysis, between CTx resistance and non-CTx resistance groups, a total of 204 DEGs were identified from GSE140973 (Additional file 3: Table S1). The top 25 up-regulated and down-regulated genes in the CTx resistance group were displayed in the heatmap (Fig. 3A). Simultaneously, the potential targets of miR-199b-3p were predicted and downloaded from Targetscan (Additional file 4: Table S2). As reported, miRNAs usually exert their roles in a variety of physiological and pathological processes by down-regulating the expression of target genes [31]. We finally obtained only one gene, CRIM1, after intersecting the down-regulated genes in CTx resistance and target genes of miR-199b-3p (Fig. 3B). Then, the interaction between miR-199b-3p and CRIM1 was verified dual-luciferase reporter assay (Fig. 3C). Since binding to Ago2 to form RNA-induced silencing complex (RISC) in the cytoplasm is required for miRNAs in regulating the expression of their target genes, FISH and anti-Ago2 RIP assays were subsequently performed to further verify our prediction. The miR-199b-3p was confirmed to be mainly expressed in the cell cytoplasm (Fig. 3D). RIP demonstrated that Ago2 was able to enrich both miR-199b-3p and CRIM1 compared with IgG (Fig. 3E), which revealed the interaction of Ago2 with both miR-199b-3p and CRIM1 in SW480-CTxR and HCT116-CTxR cells. Moreover, the protein expression level of CRIM1 was elevated by suppressing miR-199b-3p in CRC-CTxR cells, while reduced by overexpressing miR-199b-3p (Fig. 3F). These data suggested that miR-199b-3p repress translation and degrade CRIM1 in an AGO2-dependent manner by binding to CRIM1.

For the clinical samples, the protein expression level of EGFR in CRC tissues from non-responders was significantly higher than that from responders (Additional file 1: Fig. S1C). We also found that the expression of CRIM1 in CRC tissues from non-responders was significantly lower than that from responders at both mRNA and protein levels (Fig. 3G–I). More importantly, CRIM1 expression was significantly and inversely correlated with miR-199b-3p expression in the CRC tissues from non-responders (Fig. 3G). Furthermore, the analysis of CRIM1 expression in CRC-CTxR cell lines and their parental sensitive ones revealed that the down-regulation of CRIM1 was closely related to acquired resistance to CTx in CRC cells (Fig. 3J, K).

### miR-199b-3p restrains the promotion role of CRIM1 in CTx responsiveness in CRC-CTxR cells

**Fig. 4:**
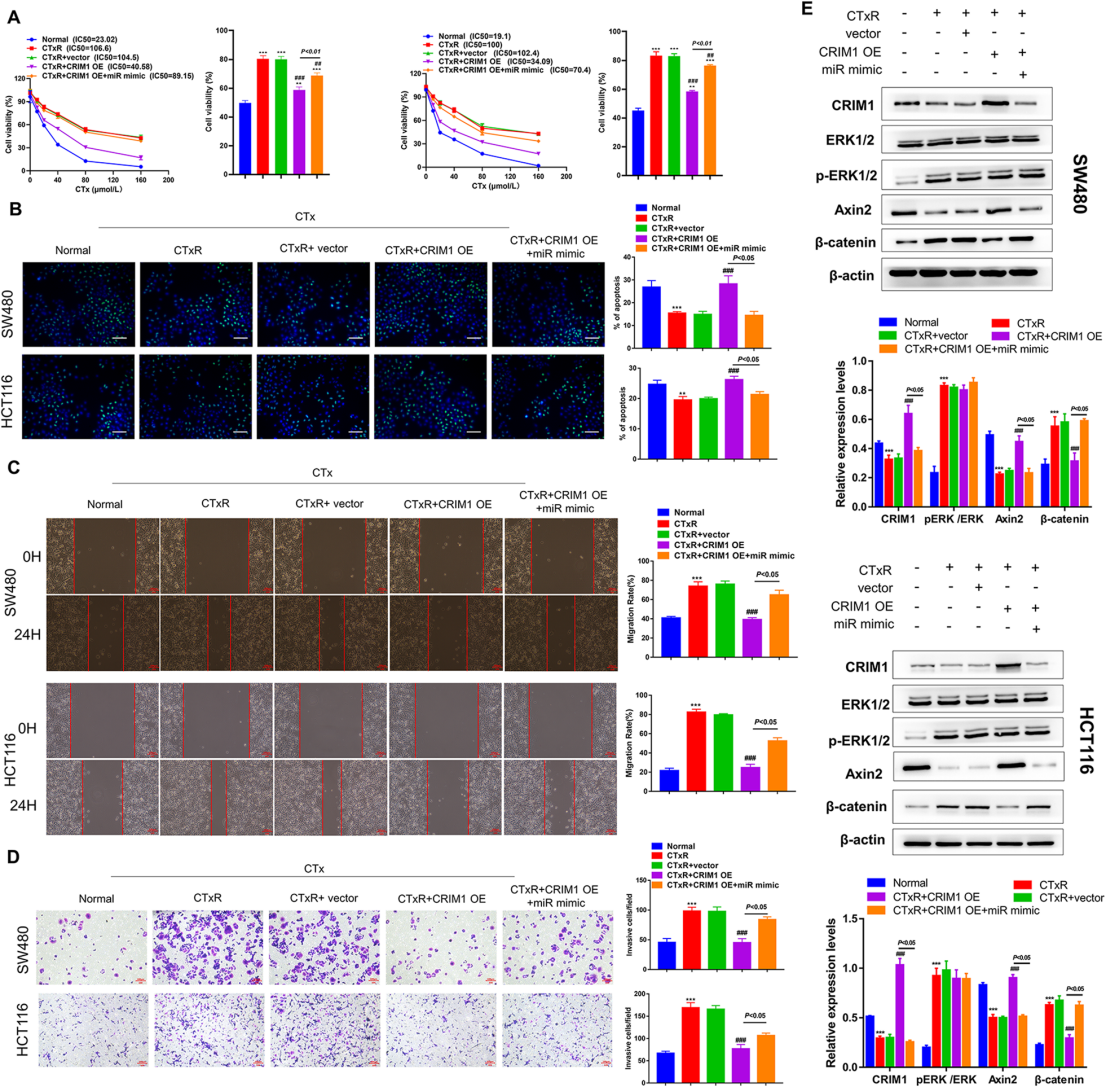
CRIM1 overexpression sensitizes CRC-CTxR cells to CTx, which was reversed by miR-199b-3p mimic. CRC-CTxR cells were transfected with CRIM1 overexpression vector (OE) alone or together with miR-199b-3p mimic (miR mimic); then, CRC-CTxR cells with or without transfection and the parental sensitive ones were treated with the same concentration of CTx. **A** CCK-8 assay analyzed the IC50 of CTx in CRC cells. **B** TUNEL assay detected the apoptotic cells of different groups. The capacity of cell migration and invasion was evaluated by **C** wound healing and **D** transwell assay, respectively. **E** Western blot detected CRIM1 and the EGFR signaling and Wnt signaling-related proteins levels. **P < 0.01, and ***P < 0.005 compared with normal group; ^##^P < 0.01 and ^###^P < 0.005 compared with CTxR group

To investigate the functional relevance of the miR-199b-3p/CRIM1 interaction on acquired resistance to CTx in CRC, we transfected CRIM1 OE alone or in combination with miR-199b-3p mimic into SW480-CTxR and HCT116-CTxR cells. Consistent with our previous experiments, the CTx responsiveness of CRC-CTxR cells was obviously weaker than that of normal CRC cells. qRT-PCR revealed that the CRIM1 OE vector is capable of up-regulating the expression of CRIM1 in CRC-CTxR cells (Additional file 1: Fig. S1D). Our study exhibited that overexpressing CRIM1 effectively enhanced the anti-tumor effects of CTx on the cell proliferation, apoptosis, migration, and invasion of CRC-CTxR cells, hinting that CRIM1 OE could reverse acquired resistance of CRC-CTxR cells to CTx (Fig. 4A–D, Additional file 2: Fig. S2B). On the other hand, these effects mediated by CRIM1 OE were partly restrained by co-transfecting with miR-199b-3p mimic (Fig. 4A–D, Additional file 2: Fig. S2B). Collectively, these data suggested miR-199b-3p contributes to acquired resistance to CTx via targeting CRIM1 in CRC cells.

Mounting evidence support that EGFR signaling and Wnt signaling are two main pathways involving in in drug resistance. To understand the regulator mechanisms of miR-199b-3p/CRIM1 axis on acquired resistance to CTx in CRC, we further detected the protein expression of ERK, Axin2, and β-catenin (Fig. 4E). In the comparison of normal CRC cells, the phosphorylation levels of ERK and β-catenin expression levels in CRC-CTxR cells were significantly increased. On the contrary, the expression levels of both CRIM1 and Axin2 in CRC-CTxR cells were significantly lower than those in normal CRC cells. When overexpressing CRIM1 in CRC-CTxR cells, the expression levels of Axin2 were significantly elevated, while those of β-catenin were reduced. However, the phosphorylation of ERK showed no change. Notably, overexpressing miR-199b-3p could reverse the effects induced by CRIM1 overexpression on the expression of Axin2 and β-catenin. Based on the above results, we postulated that miR-199b-3p/CRIM1 promotes acquired resistance to CTx in CRC cells by regulating the Wnt/β-catenin signaling pathway.

### Wnt/β-catenin signaling is essential for the promotion role of miR-199b-3p in acquired resistance to CTx in CRC cells

**Fig. 5:**
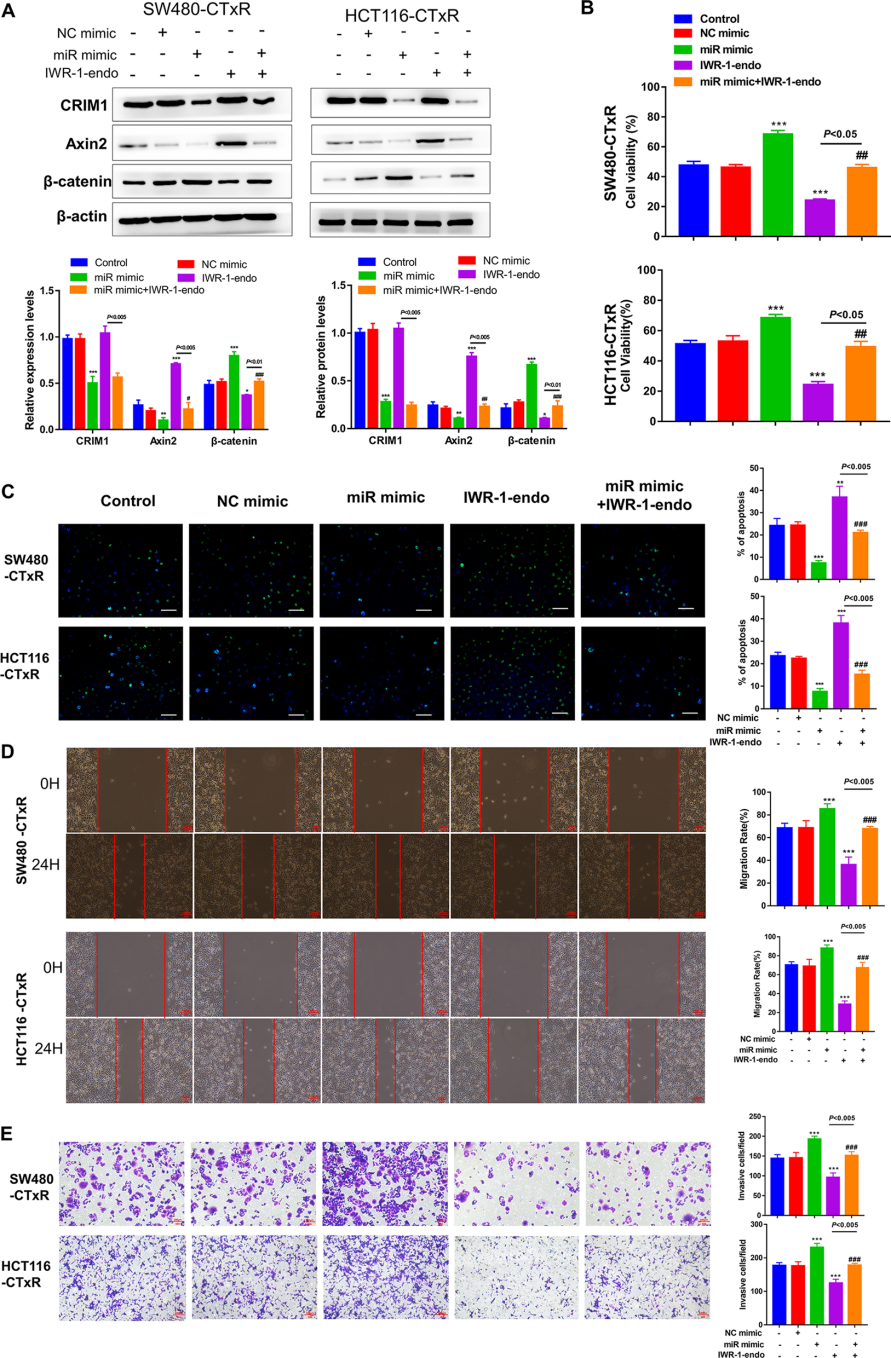
The effect of miR-199b-3p on acquired resistance to CTx was mediated by Wnt/β-catenin signaling in CRC. CRC-CTxR cells transfected with miR-199b-3p mimic (miR mimic) or NC mimic; To verify the role of miR-199b-3p in regulating Wnt signaling, miR-199b-3p overexpressing and normal CRC-CTxR cells were exposed to IWR-1-endo (Wnt signaling antagonist). **A** Western blot detected CRIM1 and the Wnt signaling-related proteins levels in CRC-CTxR cells with different treatments. **B** CCK-8 assay tested cell viability. **C** TUNEL assay detected the apoptotic cells. The capacity of cell migration and invasion was evaluated by **D** wound healing and **E** transwell assay, respectively. *P < 0.05, **P < 0.01 and ***P < 0.005 compared with control group; ^#^P < 0.05, ^##^P < 0.01 and ^###^P < 0.005 compared with miR mimic group

To corroborate that Wnt/β-catenin signaling is essential for miR-199b-3p in promoting CTx resistance in CRC cells, we introduced IWR-1-endo, a potent Wnt signaling inhibitor [32], in further analysis. The results showed that overexpressing miR-199b-3p led to a significant elevation in β-catenin expression and a reduction in both CRIM1 and Axin2 expressions (Fig. 5A). Notably, the effects of miR-199b-3p on Axin2 and β-catenin proteins were almost blocked by IWR-1-endo. Then, function analyses were conducted to investigate whether Wnt/β-catenin signaling is involved in the miR-199b-3p-induced promotion of CTx resistance. As expected, the transfection of miR-199b-3p mimic further strengthened the CTx resistance of CRC-CTxR cells, thereby increasing cell viability, migration as well as invasion, and reducing cell apoptosis under CTx treatment (Fig. 5B–E, Additional file 2: Fig. S2C). Afterward, we found that the treatment of IWR-1-endo could significantly reverse the CTx resistance promoted by miR-199b-3p, indicating that miR-199b-3p contributes to acquired resistance to CTx in CRC cells via the Wnt/β-catenin signaling pathway (Fig. 5B–E, Additional file 2: Fig. S2C).

### AntagomiR-199b-3p promotes the CTx-chemosensitivity of CRC-CTxR cells in vivo

**Fig. 6:**
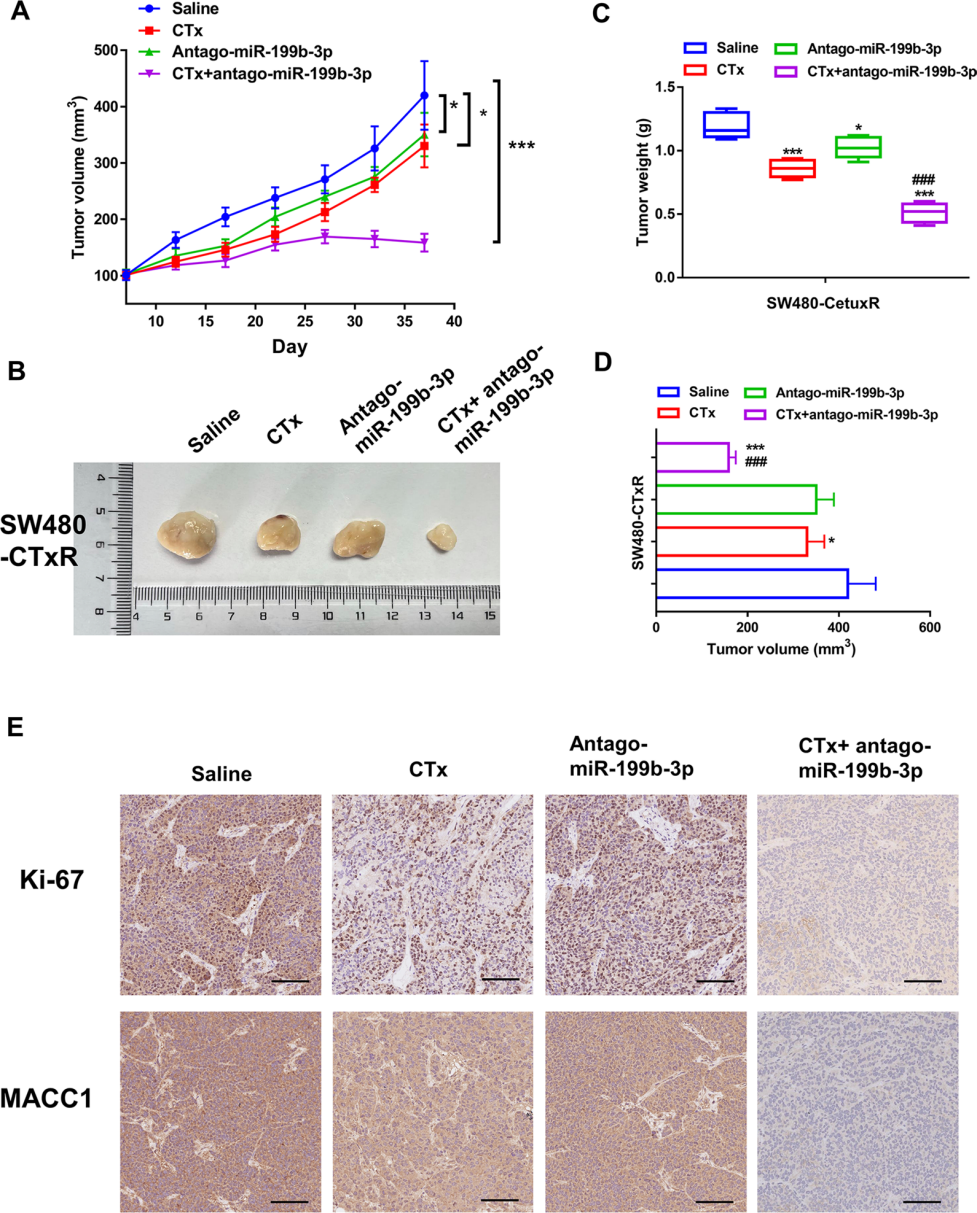
AntagomiR-199b-3p enhances the efficacy of CTx on CRC-CTxR tumor growth in vivo. After cell inoculation for a week, the volume of xenograft tumors in each group was recorded every five days (days 7, 12, 17, 22, 27, 32, and 37), **A** Growth curve and **B** representative images of xenograft tumors from each group. Tumor weights and **D** volume were recorded at the end of the experiments. **E** IHC staining determined the protein expression of Ki-67 and MACC1 in xenograft tumors (Scale bars: 100 μm). *P < 0.05 and ***P < 0.005 compared with saline group; ^###^P < 0.005 compared with CTx group

Furthermore, in vivo studies were conducted to better explore the effects of miR-199b-3p on acquired resistance to CTx. After establishing the xenograft model using SW480-CTxR cells, models from different groups were injected with saline, CTx, antagomiR-199b-3p, or CTx plus antagomiR-199b-3p. The results showed that antagomiR-199b-3p had little influence on tumorgenesis alone; however, combined with CTx, SW480-CTxR cells stably suppressing miR-199b-3p remarkably inhibited tumorigenesis, indicating antagomiR-199b-3p could enhance the efficacy of CTx to CRC in the chemoresistant context (Fig. 6A–D). Ki-67 staining was performed, which further verified that miR-199b-3p promoted proliferation (Fig. 6E). The highest positive rates of Ki-67 were observed in the saline group, followed by the antagomiR-199b-3p group, and CTx group. The CTx + antagomiR-199b-3p group represented the lowest levels of Ki-67, further indicating that the down-regulation of miR-199b-3p enhances the anti-proliferation effect of CTx in CTx-resistant tumors by inhibiting therapeutic resistance in vivo. Moreover, IHC analysis showed that the expression level of MACC1 (a novel biomarker for the prediction of metastasis for CRC) in the CTx + antagomiR-199b-3p group was higher than that in other groups, suggesting that miR-199b-3p might be involved in the metastasis of CRC (Fig. 6E). However, the role of miR-199b-3p in the metastasis of CRC is required to be further confirmed by the metastatic CRC model in vivo.

## Discussion

The development of chemoresistance is one of the challenges during CRC treatment, which eventually results in chemotherapy failure, tumor recurrence, and even patients’ death. As an FDA-approved therapy for patients with metastatic CRC, CTx has been proven to be effective in chemotherapy-refractory metastatic CRC [33]. However, acquired resistance remains a major obstacle that limited the clinical application of CTx. Hence, it is critical to identify the precise mechanisms causing acquired resistance to CTx to develop novel therapeutic strategies to achieve better clinical outcomes. Over the last decade, many miRNAs were found to be involved in the chemoresistance of CRC [34, 35]. For example, Khorrami et al. [36] reported that increased miR-146a contributed to enhancing acquired resistance to common chemotherapeutic agents (5-fluorouracil and irinotecan) in CRC cells. The current study describes the first in-depth analysis of miR-199b-3p regulatory roles in CTx resistant CRC and further explores the underlying mechanism.

In a previous study, based on miRNA microarray analysis in both CTx-resistant and -sensitive CRC cells, Mussnich et al. [14] identified a list of miRNAs that might be involved in the CTx resistance of CRC, including miR-199a-5p and miR-199b-3p. They further demonstrated that miR-199a-5p conferred CTx‐resistant properties of CRC cells through the Akt signaling pathway by targeting PHLPP1; but the roles of miR-199b-3p in CTx‐resistant are still obscure. Our study enrolled 30 CRC patients who received CTx-based chemotherapy and found that miR-199b-3p expression was significantly up-regulated in CRC tissues from patients with the progressive or stable disease compared with those with partial or complete response. Moreover, the analysis of miR-199b-3p expression in CTx-resistant CRC cell lines and their parental sensitive ones displayed a similar result to clinical samples, which is in line with the previous study conducted by Mussnich et al. [14]. Functional assays revealed that suppressing miR-199b-3p significantly sensitized CRC-CTxR cells to CTx, thereby enhancing the anti-tumor effects of CTx on CTx-resistant CRC in vitro and in vivo. These data demonstrated that miR-199b-3p may play a role in acquired resistance to CTx of CRC.

Mounting studies demonstrated that miRNAs exert their regulatory roles in various diseases by hampering the expression of target genes via binding to their 3’-UTR [35, 37]. It has been reported that miR-130b suppresses its target PTEN's expression to activate Wnt/β-catenin pathway thereby inducing resistance to cisplatin in lung cancer cells [38]. A recent study demonstrated that miR-221-3p confers drug resistance of breast cancer cells to adriamycin by targeting PIK3R1 in vitro and in vivo [39]. Based on bioinformatics analysis, we identified CRIM1 as the potential target of miR-199b-3p in CTx-resistant CRC. Nakashima et al. [40] demonstrated that the up-regulation of CRIM1 can repress not only the proliferation but also the migration ability of vascular endothelial cells. Additionally, Ogasawara’s group [41] found that CRIM1 plays a suppression role in the migration and invasion of renal carcinoma cells via regulating EMT-related factors. These literatures signposted the anti-cancer effect of CRIM1. By luciferase reporter assay, anti-Ago2 RIP assay, and qRT-PCR, we confirmed that miR-199b-3p directly interacts with CRIM1 in a physical way. Both qRT-PCR and western blot analysis on CRC tumors revealed CRIM1 is lowly expressed and negatively correlated with miR-199b-3p level in CTx resistant CRC tissues. Moreover, overexpressing miR-199b-3p led to a significant decrease in the expression of CRIM1 in SW480-CTxR/HCT116-CTxR cells, and vice versa. These data collectively indicated that miR-199b-3p bound directly to CRIM1 3ʹUTR and regulated the post-transcriptional expression of CRIM1 in SW480-CTxR/HCT116-CTxR cells. Further in vitro functional analyses demonstrated that increased CRIM1 restored the responsiveness of SW480-CTxR/HCT116-CTxR cells; but this effect was blocked by overexpressing miR-199b-3p, which provided evidence that CRIM1 worked as not only downstream target but also the mediator of miR-199b-3p in the resistance of SW480-CTxR/HCT116-CTxR cells to CTx.

Multiple studies documented that abnormal Wnt signaling serves an important role in CRC, which is close to tumor growth, metastasis, as well as chemoresistance [42]. Therefore, our study further explored whether the role of CRIM1/miR-199b-3p in CTx-resistance is exerted by regulating Wnt signaling. In this study, the expression levels of β-catenin in CRC-CTxR cells were obviously up-regulated compared with those in normal CRC cells, demonstrating the activation of the canonical Wnt-signaling pathway in CRC-CTxR cells. Moreover, the down-regulation of Axin2 in CRC-CTxR cells corroborated this finding. As known, β-catenin is an important regulatory molecule of the canonical Wnt-signaling pathway, of which phosphorylation level is inversely correlated with the Wnt activation and modulated by the destruction complex composing of APC, Axin, and GSK3β [17]. When overexpressing CRIM1 in CRC-CTxR, the activation of Wnt-signaling was potently blocked. In the meantime, overexpressing miR-199b-3p displayed the opposite effect on this pathway in CRC-CTxR cells, suggesting miR-199b-3p confers chemoresistance to CRC cells through activating Wnt signaling via CRIM1 suppression. The further functional analysis showed that the inhibitor of Wnt/β-catenin (IWR-1-endo) could almost block the CTx-resistance induced by miR-199b-3p, verifying our finding that miR-199b-3p contributes to acquired resistance to CTx in CRC through regulating Wnt/β-catenin signaling. Collectively, our data revealed that miR-199b-3p is up-regulated in CRC-CTxR cells and tissues, and that it is responsible for acquired resistance to CTx in CRC. Mechanistically, miR-199b-3p up-regulation activates Wnt/β-catenin signaling through suppressing CRIM1 expression, thereby driving CTx resistance in CRC. Combined with the results of previous studies implicating the indirect interaction of the cytoplasmic domain of CRIM1 with β-catenin during nervous system development [41], it may be inferred that CRIM may impede Wnt signaling by binding to β-catenin during cancer progression. However, the precise mechanism that how CRIM1 acts on Wnt/β-catenin signaling in acquired resistance to CTx in CRC is required for an in-depth study in the future.

## Conclusions

In summary, the current study demonstrated that high miR-199b-3p expression is associated with acquired resistance to CTx in CRC and that the CRIM1/Wnt/β-catenin signaling is engaged in miR-199b-3p-mediated CTx resistance. These findings suggest that miR-199b-3p might represent a promising therapeutic target against CTx resistant CRC, providing scientific information for exploring novel strategies for improving the efficacy of CTx for CRC patients.

## Supplementary Information

**Additional file 1: Figure S1.** The expression level of EGFR and the confirmation of transfection efficiency on CRC-CTxR cells. (A) Western blot detected the expression of EGFR between CRC-CTxR cell lines and the parental sensitive ones. (B) qRT-PCR verified the successful transfection of miR-199b-3p inhibitor or mimic. (C) Western blot detected the expression of EGFR in CRC tissues from responder and non-responder. (D) qRT-PCR verified the successful transfection of CRIM1 overexpression vector. Note: ^***^P < 0.005 compared with NC inhibitor, NC mimic, or vector group.

**Additional file 2: Figure S2.** The effect on the apoptosis of CRC-CTxR cells. Western blot detected the expression of apoptosis-related proteins (Bax and Bcl-2; β-actin considered as loading control).

**Additional file 3: Table S1.** The DEGs between CTx resistance and non-CTx resistance groups in the GSE140971 dataset.

**Additional file 4: Table S2.** The potential targets of miR-199b-3p predicted by Targetscan.

**Additional file 5: Table S3.** The usage information of antibodies in this study.

## Data Availability

Not applicable.

## Abbreviations

CRC: Colorectal cancer
MiRNAs: MicroRNAs
CTx: Cetuximab
CTxR: CTx-resistant
EMT: Epithelial-mesenchymal transition
APC: Adenomatous polyposis coli
GSK3β: Glycogen synthase kinase 3β
TCF/LEF: T-cell factor/lymphoid enhancer binding factors
RECIST: Response evaluation criteria in solid tumors
FISH: Fluorescence in situ hybridization
IHC: Immunohistochemistry
qRT-PCR: Quantitative real-time PCR
OD: Optical density
TdT: Terminal deoxynucleotidyl transferase
DEGs: Differentially expressed genes
FC: Fold change
MUT: Mutation
RIP: RNA immunoprecipitation
