# Supplementary figures and images for: miR-199b-3p contributes to acquired resistance to cetuximab in colorectal cancer by targeting CRIM1 via Wnt/β-catenin signaling
Source: Cancer Cell Int. 2022 Jan 28;22:42. doi: 10.1186/s12935-022-02460-x (PMC8796585; doi:10.1186/s12935-022-02460-x)

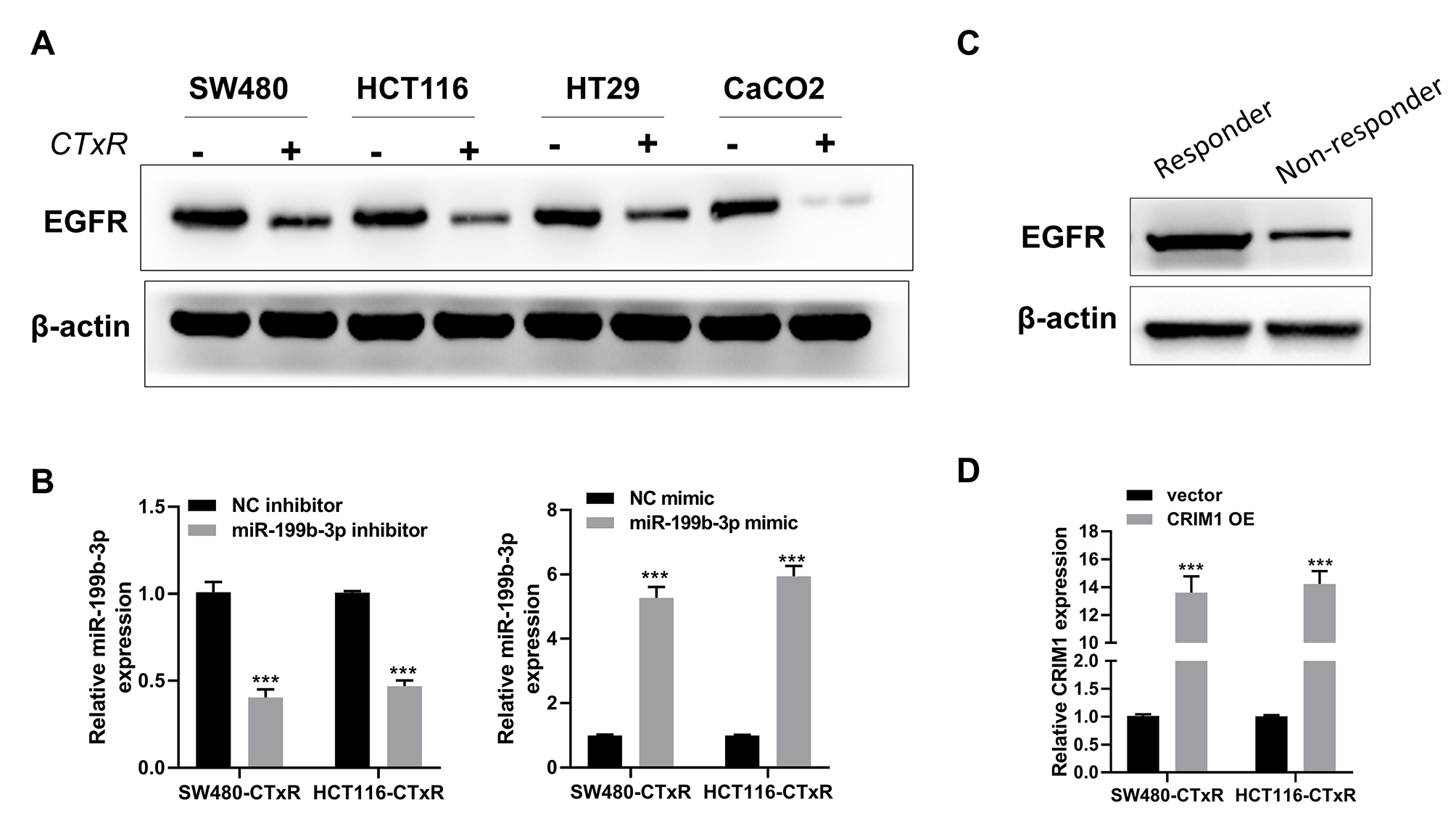

Supplement: Supplementary file 1 — Additional file 1: Figure S1. The expression level of EGFR and the confirmation of transfection efficiency on CRC-CTxR cells. (A) Western blot detected the expression of EGFR between CRC-CTxR cell lines and the parental sensitive ones. (B) qRT-PCR verified the successful transfection of miR-199b-3p inhibitor or mimic. (C) Western blot detected the expression of EGFR in CRC tissues from responder and non-responder. (D) qRT-PCR verified the successful transfection of CRIM1 overexpression vector. Note: ***P < 0.005 compared with NC inhibitor, NC mimic, or vector group. [file 12935_2022_2460_MOESM1_ESM.tif]

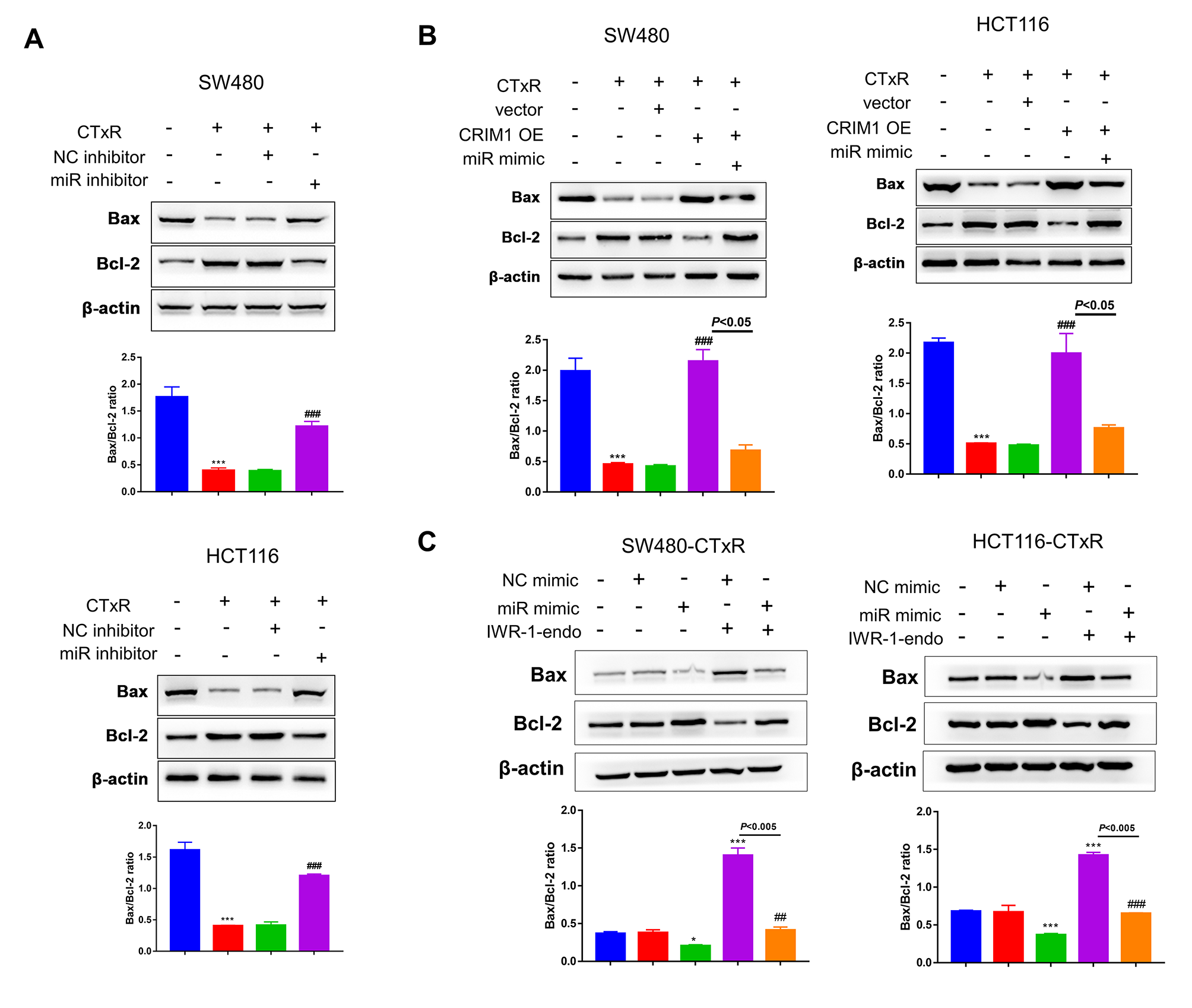

Supplement: Supplementary file 2 — Additional file 2: Figure S2. The effect on the apoptosis of CRC-CTxR cells. Western blot detected the expression of apoptosis-related proteins (Bax and Bcl-2; β-actin considered as loading control). [file 12935_2022_2460_MOESM2_ESM.tif]
