# Supplementary material for: miR-199b-3p contributes to acquired resistance to cetuximab in colorectal cancer by targeting CRIM1 via Wnt/β-catenin signaling
Source: Cancer Cell Int. 2022 Jan 28;22:42. doi: 10.1186/s12935-022-02460-x (PMC8796585; doi:10.1186/s12935-022-02460-x)
Supplement: Supplementary file 5 — Additional file 5: Table S3. The usage information of antibodies in this study. [file 12935_2022_2460_MOESM5_ESM.docx]

**Table S3. The usage information of antibodies in this study**

|  | Application | Titration | Dilution |
| --- | --- | --- | --- |
| CRIM1 | IHC-P | 1:800 | 1:500 |
|  | WB (tissue) | 1:1200 | 1:1000 |
|  | WB (cell) | 1:1000 | 1:1000 |
| Ki-67 | IHC-P | 1:2000 | 1:500 |
| MACC1 | IHC-P | 1:250 | 1:200 |
| Bax | WB | 1:10000 | 1:8000 |
| Bcl-2 |  | 1:2000 | 1:2000 |
| ERK1/2 |  | 1:1000 | 1:800 |
| p-ERK1/2 |  | 1:1000 | 1:800 |
| Axin2 |  | 1:2000 | 1:1500 |
| β-catenin |  | 1:10000 | 1:8000 |
| β-actin | WB (tissue) | 1:800 | 1:500 |
|  | WB (cell) | 1:1000 | 1:800 |
| Goat Anti-Human IgG H&L (HRP) | IHC-P | 1:1000 | 1:1000 |
| Goat Anti-Mouse IgG+IgM H&L (HRP) | IHC-P | 1:2500 | 1:2000 |
| Goat Anti-Rabbit IgG H&L (HRP) | WB | 1:20000 | 1:5000 |
